# Network cloning using DNA barcodes

**DOI:** 10.1073/pnas.1706012116

**Published:** 2019-04-24

**Authors:** Sergey A. Shuvaev, Batuhan Başerdem, Anthony M. Zador, Alexei A. Koulakov

**Affiliations:** ^a^Cold Spring Harbor Laboratory, Cold Spring Harbor, NY 11724

**Keywords:** neural networks, connectomics, DNA barcodes, neural development

## Abstract

The connections between neurons determine the computations performed by both artificial and biological neural networks. Recently, we have proposed SYNSeq, a method for converting the connectivity of a biological network into a form that can exploit the tremendous efficiencies of high-throughput DNA sequencing. In SYNSeq, each neuron is tagged with a random sequence of DNA—a “barcode”—and synapses are represented as barcode pairs. SYNSeq addresses the analysis problem, reducing a network into a suspension of barcode pairs. Here, we formulate a complementary synthesis problem: How can the suspension of barcode pairs be used to “clone” or copy the network back into an uninitialized tabula rasa network? Although this synthesis problem might be expected to be computationally intractable, we find that, surprisingly, this problem can be solved efficiently, using only neuron-local information. We present the “one-barcode–one-cell” (OBOC) algorithm, which forces all barcodes of a given sequence to coalesce into the same neuron, and show that it converges in a number of steps that is a power law of the network size. Rapid and reliable network cloning with single-synapse precision is thus theoretically possible.

## Significance

The connections between neurons determine the computations performed by a neural network. Connections can be considered a “summary” of the statistical structure of the experience—data—on which the network was trained. Here, we propose a method for how neuronal network connectivity can be copied or “cloned” from one network to another. Our method relies on the use of DNA barcodes—short DNA sequences that allow tagging individual neurons with unique labels. In our study, we prove theorems that show that such a transfer of network connectivity is theoretically possible.

The connections between neurons determine the computations performed by a neural network. In both biological and artificial neural networks, connections are established and tuned by experience and learning. Connections can thus be considered a “summary” of the statistical structure of the experience—data—on which the network was trained. This summary may be considerably more compact and efficient than the original data. For example, deep neural networks for object recognition contain tens of millions of connections derived from training sets consisting of hundreds of billions pixels, which results in more than 1,000-fold compression (1, 2). It would therefore be more efficient to copy these connections onto a new network than to retrain a new network from scratch.

Most current implementations of artificial neural networks exploit digital computers and graphics processing units (2). On these architectures, connections are stored explicitly and are therefore straightforward to extract and copy into a new network. In biological networks, by contrast, there is no central repository for connections, so reading out the connections of a network and copying them into a new network represents a difficult challenge. During neural development, for example, a genomic DNA sequence representing prior evolutionary experience is converted into the brain’s connectivity. Similar challenges may arise in future artificial or hybrid biological/artificial architectures.

**Fig. 1.**
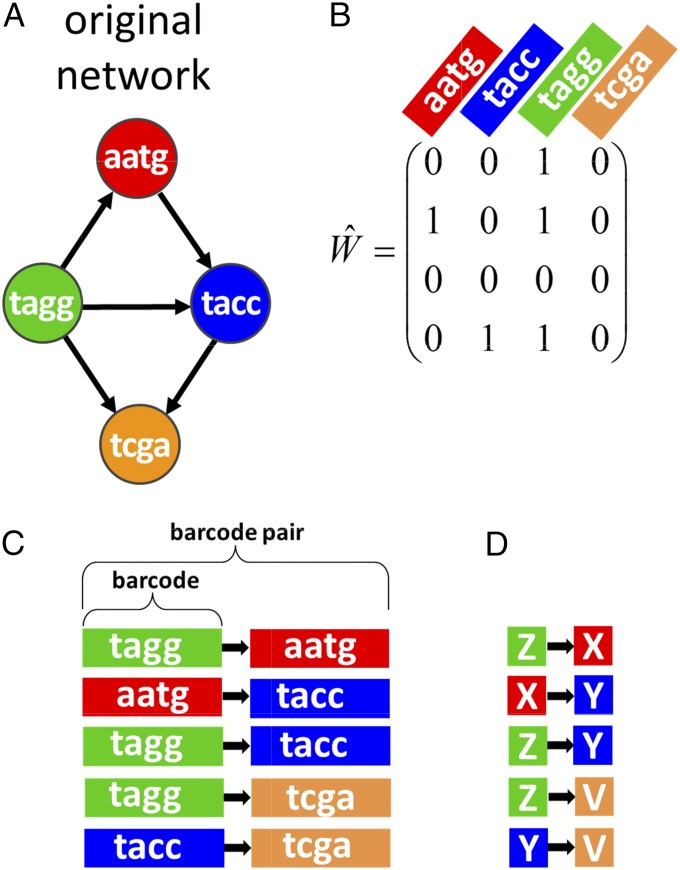
Representation of a network by an ensemble of barcode pairs (SYNSeq). (*A*) An example of small network. In SYNSeq, each neuron is represented by a short unique nucleotide sequence called a barcode. (*B*) The connectivity matrix corresponding to the network in *A*. (*C*) Network connections are encoded by pairs of barcodes with a spacer (black arrow) representing the connections’ direction. (*D*) We represent barcodes by unique letters of an alphabet for brevity.

We have recently proposed SYNSeq, an approach for determining neuronal connectivity (3, 4). The key idea is to convert the connections into a form that can be read out using high-throughput DNA sequencing, thereby benefitting from the advances in sequencing technology. Sequencing is now extremely fast and inexpensive—it is routine to decode billions of DNA fragments per day, and sequencing cost has dropped at a rate faster than Moore’s law. To convert neuronal connectivity into a sequencing problem, we induce individual neurons to express unique random nucleotide identifiers called “barcodes.” Pairs of presynaptic and postsynaptic barcodes represent individual synaptic connections. These barcode pairs can then be used to represent the connectivity of a network (Fig. 1).

Here, we formulate a different problem: Given an ensemble of connections represented by barcode pairs, can we copy them into a new network? In other words, can the original network be cloned? We explore a computational model that simulates the behavior of barcodes introduced into a tabula rasa network with unstructured connectivity and test its ability to recreate target connectivity in such networks. We require the underlying mechanisms to be purely local, that is, that the algorithm uses only information available to a given neuron and its synapses. Below, we present an algorithm that allows robust copying of connectivity based only on local interactions.

In our approach, connectivity is specified by unique molecular labels (DNA barcodes) with single-synapse precision. It is commonly assumed that implementing connectivity via individual synaptic tags is not feasible due to the absence of guidance mechanism that would direct the cells to form the right synapses (5). One might expect that establishing desired connectivity using individual synaptic labels would require a number of steps that is exponential in network size. The inadequacy of unique molecular tags in instructing connectivity had motivated Roger Sperry (6) to introduce the idea of molecular gradients. Here, we propose a form of molecular dynamics and find, surprisingly, that it yields convergence to the target connectivity in a number of steps that is polynomial in network size, even though the connectivity is specified by unique molecular labels for each synapse. This finding implies that copying connectivity with single-neuron precision using our strategy is theoretically possible.

## Results

Our algorithm attempts to recreate the target connectivity between neurons (Fig. 1*A*). The connectivity can be represented as a connection matrix $\hat{W}$ (Fig. 1*B*). We assume that every network node (neuron) is identified by a unique barcode, that is, by a sequence of nucleotides long enough to label uniquely every neuron in the network (Fig. 1*A*). Network connectivity is thus encoded by barcode pairs, where each barcode pair consists of a presynaptic barcode, a postsynaptic barcode, and a spacer between them indicating the connection’s direction (Fig. 1*C*). This network description is similar to the netlist representation (7). As seen in Fig. 1*C*, every barcode, representing an individual neuron, can be encountered multiple times, equal to the number of synapses made by this neuron. We therefore define a barcode type as the set of barcodes with the same sequence that represents the same neuron. Each barcode pair, on the other hand, is present in only one copy, because it represents an individual synaptic connection. The total number of barcode pairs is equal to the number of nonzero entries in the connection matrix, or to the total number of connections in the network. To simplify notation, we will represent each barcode by a single letter of the alphabet rather than as a string of nucleotides (Fig. 1*D*).

**Fig. 2.**
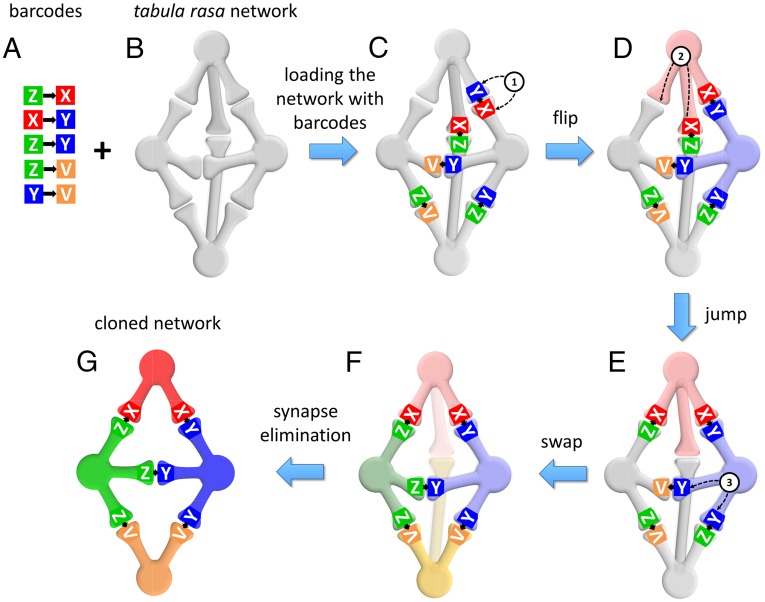
The “one-barcode–one-cell” (OBOC) rule yields target connectivity. (*A*) The set of barcode pairs representing the original network from Fig. 1. Individual barcode sequences are shown as letters for brevity. Barcode pairs represent individual synapses. (*B*) An all-to-all connected tabula rasa network that receives the ensemble of barcode pairs. We show connections as undirected synapses for simplicity. (*C*) The barcode pairs are initially arranged randomly. (*D*–*F*) Barcode pairs can move through the network by jumping from synapse to synapse using three moves as illustrated: flips (*i*), jumps (*ii*), and swaps (*iii*). The moves minimize the cost function defined by Eq. **1**. (*F*) Minimization of the cost function forces all barcodes facing every neuron to be the same. This arrangement is called OBOC. Once OBOC solution is achieved, we eliminate all synapses that contain no barcode pairs, such as the synapse between cells “X” and “V.” (*G*) OBOC solution yields the copying of the original connection matrix.

The barcode pairs are introduced into synapses of a tabula rasa network that is, initially, fully connected (Fig. 2*B*). Since connectivity in our model is directional, we assume that, between every two cells, synapses are formed initially in both directions. The full connectivity assumption is made here to simplify the description of network dynamics, and is reminiscent of the overproduction of synaptic connections that occurs during development (8, 9) and the full potential cortical connectivity (10). The number of neurons in the tabula rasa network is assumed to be equal to the number of nodes in the desired network, that is, equal to the number of barcodes.

The barcodes are initially introduced into synapses of tabula rasa network randomly. One possible solution for recreating the target network is to mark each neuron of the tabula rasa network with individual barcode tags and to distribute the barcode pairs into synapses according to these tags. The unoccupied synapses of the tabula rasa network would subsequently be eliminated. Implementing this mechanism practically would require a global supervising mechanism that keeps track of unique label assignments and appropriate barcode placements. Such mechanism is therefore not biologically plausible. Instead, here we formulate a fully local procedure for recreating the target network in the tabula rasa network in which processes in each cell rely only on the information available to each cell. Thus, the target network emerges as a result of self-organization of barcodes in the tabula rasa. The barcodes are rearranged in the network via three types of local moves. First, each barcode can be reinserted in the synapse between the same pair of cells in different orientation (“flips”; Fig. 2*C*). Second, the barcodes can jump from one synapse to another synapse of the same cell (“jumps”; Fig. 2*D*). Finally, two barcodes located in the same neuron can trade places (“swaps”; Fig. 2*E*). To practically implement these three moves, we select two synapses of the same neuron at random, ensure that at least one of them contains a barcode pair, and swap the pairs, even if source and destination are the same or one of them is empty. In implementing these moves, we keep track of the direction of barcode pairs and synapses, that is, barcode pairs are introduced into synapses of the correct orientation. We ensure that the described moves are local in that the barcode pairs are only relocated between synapses of the same neuron.

Using this set of moves, we rearrange barcode pairs in the network attempting to implement the “one-barcode–one-cell” (OBOC) solution. In the OBOC solution, all barcodes in the synapses of the same cell, facing this cell, are the same (Fig. 2*G*). Thus, in Fig. 2*G*, all barcodes in the rightmost cell are described by letter Y (V, X, Y, Z is a short-hand notation for much longer nucleotide sequences). Similarly, all barcodes in the leftmost cell are labeled by letter Z. We reasoned that if the logic of the interaction of cells and barcodes favors OBOC solution, cells will discover their identity as encoded by barcodes. Because every cell in the tabula rasa network has a potential to become any cell as defined by the barcodes, a specific arrangement of barcode pairs respecting OBOC rule is associated with a symmetry breaking, whereby the network selects one possible assignment of barcodes into cells out of N! combinations (N is the number of neurons in the network, and is equal to the number of barcodes). We also reasoned that if we then eliminate all synapses that are not occupied by a barcode pair, the remaining synapses will implement the target connectivity.

To practically implement OBOC solution, we defined a cost function, H, that is minimized by the barcode dynamics. The cost function depends on the synapse–barcode connection index (SBCI), $x_{ij,\nu \mu }$, which determines which barcode pair is present in what synapse. This variable is equal to 1 or 0 if a barcode pair connecting two barcodes μ→ν is present or absent in a synapse from cell j to i (μ, ν, i, and j are unique indexes enumerating barcodes and cells). The constraint on SBCI is that after summing it over all synapses, we should obtain the original barcode connectivity matrix such as the one shown in Fig. 1*B*: $\sum_{ij}x_{ij,v\mu }=W_{v\mu }$. Index $c_{n\beta }$ defines the number of barcodes (not pairs) of type β in cell n. To find this number, for a given cell n and barcode type β, we have to sum SBCI over all other cells in the network and all other barcode types represented by indexes m and α, respectively, that is, $c_{n\beta }=\sum_{m\mu }x_{nm,\beta \mu }+\sum_{m\mu }x_{mn,\mu \beta }$. This equation includes two contributions, because, in our case, synapses are directional and contributions from both n→m and m→n have to be counted. Although many choices are possible for the cost function, we use this particular form:

$$H=-\left(1+\varepsilon \right)\sum_{n=1}^{N}\sum_{\beta =1}^{B}{\left(c_{n\beta }\right)}^{\gamma }+\varepsilon \sum_{n=1}^{N}(\sum_{\beta =1}^{B}{c_{n\beta })}^{\gamma }.$$ [1]

Here, sums are assumed over the neuronal index n ranging from 1 to N, the total number of neurons, and the barcode index β, ranging from 1 to B, the total number of barcodes. γ and ε are the parameters of the cost function. To implement the OBOC rule, the parameter γ must exceed unity. In the present work, we used γ=2, in which case the first term of the cost function can be viewed as the measure of the sparseness of barcode distribution $c_{n\beta }$ (11–15). Minimization of this term leads to a sparser distribution $c_{n\beta }$, that is, the distribution that has more zeros values. As a consequence, the first term in the cost function achieves its minimum when barcodes of the same type reside in the same neuron, that is, when OBOC solution is reached. For example, if two copies of the barcode of the same type are present and if they are located in the same cell, the first term of the cost function is equal to $-\left(1+\varepsilon \right)2^{2}=-4\left(1+\varepsilon \right)$ for γ=2. If these two barcodes are distributed between two different cells, the first term in the cost function is $-\left(1+\varepsilon \right)\left(1^{2}+1^{2}\right)=-2\left(1+\varepsilon \right)$. Thus, minimizing the first term favors the convergence of the barcodes of the same type in the same cell. This alone, however, does not prevent barcodes of different types from congregating in the same cell. To prevent this from happening, we introduced the second term into the cost function. Minimizing second term leads to the separation of the barcodes of different types into different cells. Assume that two types of barcodes are present in the system, with two copies each. The values of the second term when these two barcodes types are placed into the same or different cells are $4^{2}\varepsilon$ and $\left(2^{2}+2^{2}\right)\varepsilon$, respectively, leading to the desired effect. Parameter ε, which we set to ε=10, determines the balance between two terms of the cost function. For γ=2, the cost function can be written as $H=\sum_{n=1}^{N}{\vec{c}}_{n}^{T}\hat{U}{\vec{c}}_{n}$, where ${\vec{c}}_{n}$ is the vector of barcode abundances in neuron number n, and $\hat{U}=-\left(1+\varepsilon \right)\hat{I}+\varepsilon \hat{Y}$. Here, $\hat{Y}$ is the matrix of all ones. Because the diagonal part of matrix $\hat{U}$ $\left(∼\hat{I}\right)$ is negative, it favors solutions in which there is a single barcode type per cell, while the off-diagonal part $\left(∼\hat{Y}\right)$ penalizes multiple barcode types in a cell. It therefore represents the repulsion of different barcodes present in the same cell. Both of these components help achieve OBOC solution.

Importantly, the cost function [**1**] has a property of locality, that is, the contribution for each neuron depends on the variables available to this neuron $c_{n\beta }$, that is, the number of barcodes of type β. The decision whether the barcode move lowers the cost function, and as consequence, whether such a move should be implemented, depends on the information available to the cell and its synapses only. Minimizing the cost function [**1**] does not require a global supervisor, which would render the mechanism biologically implausible.

The approach based on minimizing a cost function is one of the ways to quantitatively describe biological processes and has been used successfully to describe establishing connectivity, especially when competition or interdependence between cells is important (16, 17). To minimize the cost function, we use the Metropolis Monte Carlo (MMC) procedure that has been shown to closely approximate the dynamics of synaptic connections during neural development (8, 16–18). Our MMC procedure relied on three types of barcode moves as described above. After the cost function is minimized, at the end of the MMC procedure, we remove synapses that carry no barcodes. Within our model, we can prove the following theorems with regard to reproducing the target connectivity. The detailed proofs are provided in *Appendix*.

### Theorem 1.

*Let* ${\hat{W}}_{B}$ *be the target connectivity defined by the barcode pairs*. *Let* ${\hat{W}}_{N}$ *be a cell connectivity corresponding to an OBOC solution for the same set of the barcode pairs arising after barcode-free synapses are eliminated*. *Then*, *a one-to-one mapping* $\hat{M}$ *exists between the set of barcodes and the neurons*, *which makes* ${\hat{W}}_{B}=\hat{M}{\hat{W}}_{N}{\hat{M}}^{T}$.

*Theorem 1* shows that reaching OBOC state is equivalent to cloning the target connectivity. Although this statement is quite obvious, we prove it in *Appendix* for completeness. In *Appendix*, we show that connectivity can be cloned up to a permutation with $\hat{M}$ being a permutation matrix. The problem of network copying therefore has N! equivalent (isomorphic) OBOC solutions.

### Theorem 2.

*For* γ=2 *and* ε≥1, *in a non-OBOC state*, *there is always a barcode jump decreasing the cost function*.

*Theorem 2* shows that the cost function [**1**] does not have any non-OBOC minima, meaning that the barcode dynamics would not lead to a metastable, yet wrong, connectivity. Therefore, we prove the following corollary.

### Corollary.

*For* γ=2 *and* ε≥1, *all of the minima of the cost function* [**1**] *correspond to OBOC solutions*.

This corollary combined with the theorems above shows that minimization of the cost function [**1**] will lead to an OBOC solution, thus cloning the barcode defined connectivity in the network. To estimate the number of steps until convergence to the target connectivity, we proved the following theorem.

### Theorem 3.

*For* γ=2 *and* ε≥1, *convergence to a minimum of the cost function* [**1**] *takes a number of steps limited from above by a number polynomial in the number of network nodes* (*cells*). *Theorem 3* shows that the convergence of the network connectivity to the target is not exponential.

**Fig. 3.**
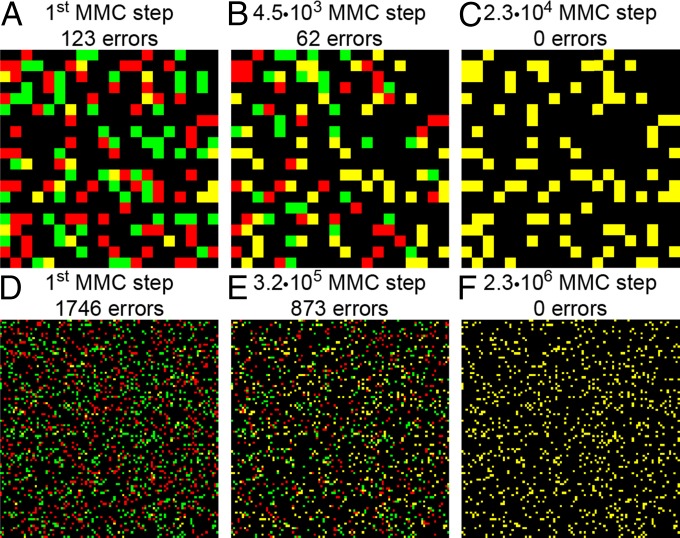
The OBOC rule allows copying desired network connectivity matrix. Results of a single MMC run for 20 × 20 (*A*–*C*) and 100 × 100 (*D*–*F*) networks. Red/green channels show target/actual connection matrices. Yellow matrices at the end of the simulation run (*C* and *F*) indicate a perfect copy.

**Fig. 4.**
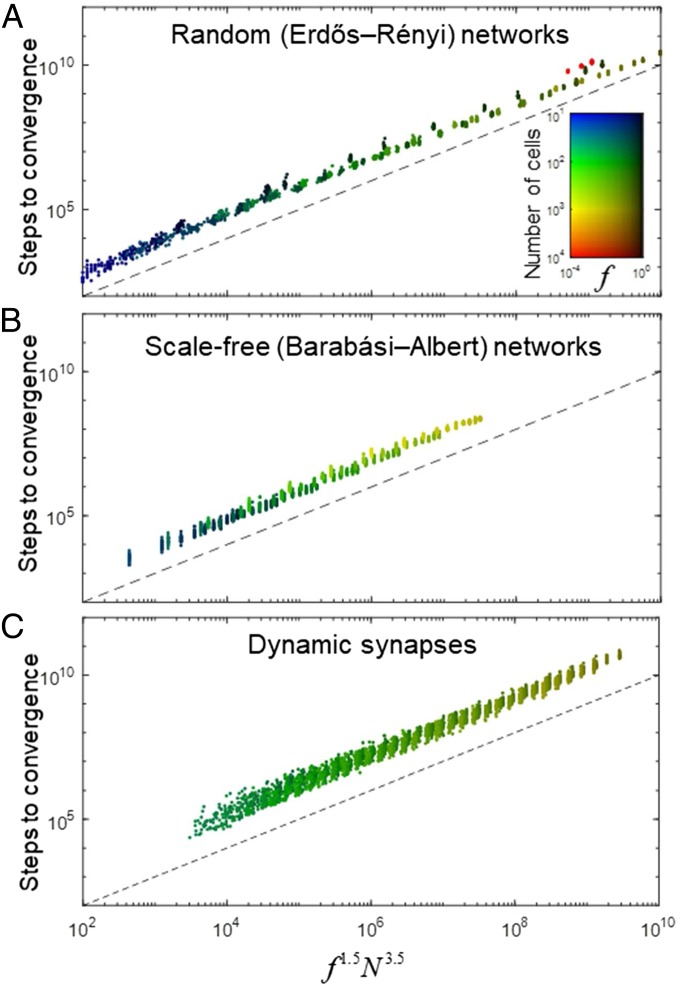
OBOC rule yields target connectivity in a number of steps given by a power law of network size. Number of steps required for convergence as a function of the combination of network parameters $f^{1.5}N^{3.5}$ (N, the size of the network, and f, the fraction of nonzero connections). Network size varied between N=10 and $10^{4}$. Each point represents an individual simulation. Individual network parameters are identified by color for each simulation. Dashed lines represent the number of steps given by the identity $N_{\text{steps}}=f^{1.5}N^{3.5}$. (*A*) Random (Erdős–Rényi) target connectivity. (*B*) Scale-free (Barabási–Albert) target connectivity. (*C*) Synaptic dynamics: Every fixed number of steps, we relocated all of the empty synapses (∼100 times during the entire simulation, on average).

To measure the number of steps needed to clone network connections and to obtain a stronger estimate for the speed of convergence to the target connectivity under various circumstances, we performed several computer simulations (Fig. 3). To generate the examples of target connectivities, we used random networks of various topologies, connectivity density f, and size N. To quantify the speed of convergence, for each MMC simulation, we computed the number of attempts, $N_{\text{steps}}$, to move the barcodes before a perfect OBOC solution was achieved (Fig. 4). We found that the number of steps is well approximated by a power law:

$$N_{\text{steps}}\propto f^{1.5}N^{3.5}.$$ [2]

The power law [**2**] holds for both random Erdős–Rényi (19) networks (Fig. 4*A*) and scale-free (Barabási–Albert) (20) networks (Fig. 4*B*), suggesting that the dependence of the performance on the network topology is negligible compared with the dependence on the connectivity density and network size.

So far, we have assumed that target networks initially have an all-to-all connectivity. Barcode-free synapses are eliminated at the end of cost-function minimization, after the barcode pairs have found an OBOC solution. This full initial connectivity assumption is clearly a simplification intended to mimic the overproduction of synapses during neural development (8, 9). In reality, the formation of connectivity is accomplished via the process of trial and error during which synapses are both created and eliminated (8, 9). To test our conclusions in the model in which synapses can be formed and pruned while the barcode pairs are relocated in the network, we performed simulations with the same cost function in the conditions when synaptic connectivity is both sparse and dynamic. Initially, tabula rasa network was sparse, with the sparseness parameter exceeding the sparseness of the barcode matrix. The number of excess synaptic connections was given by the formula $0.3\left(1-f\right)N^{2}$ (see *Methods* for more detail). Thus, for f≈0 and f=1, the numbers of excess synapses were $0.3N^{2}$ and 0, respectively, while changing linearly between these values. One hundred times during each simulation, the synapses lacking any barcode pairs were relocated randomly to pairs of cells that were not at the moment connected. At the end of the simulation, empty synapses were eliminated as in the case of static network. We found that a similar power law for the convergence [**2**] holds in the case of dynamic synapses (Fig. 4*C*), meaning that the network should not be necessarily fully connected to obtain an accurate copy of the connectivity in a polynomial number of steps.

## Discussion

Here, we have addressed the question whether connectivity can be copied from one neural network to another, using only a local rule. It should be noted that the connectivity in the original network can be obtained using any paradigm that results in connection matrix with the single-synapse precision, such as using volume electron microscopy methods (21, 22) or SYNSeq approach (3, 4). Original connectivity can also result from an application of a learning algorithm in an artificial neural network (1, 2). Independently on their origin, the connections can be represented by an ensemble of DNA barcode pairs (3, 4). We analyzed the dynamics of barcode pairs introduced into a clean-slate tabula rasa network. The particular form of dynamics that we considered is described by OBOC, which favors positioning of a single type of barcodes in a single neuron. We showed that OBOC dynamics leads to fast and reliable recreation of desired connectivity in the new network. The formation of new connectivity is achieved in a number of steps given by a power law of the network size [**2**]. We have proved a convergence theorem (*Theorem 2*) showing that movements of barcodes toward OBOC solution are not obstructed by local minima. Thus, we have demonstrated that copying connectivity from one neural network to another using DNA barcodes is theoretically possible.

The number of steps to convergence, defined by Eq. **2**, may seem impractical, as the number of steps grows rapidly with the network size. Using only local information in cost function [**1**], however, allows moving the barcode pairs in parallel, thus reducing the number of the steps to the convergence. Since the number of barcode pairs is $B=fN^{2}$, the number of attempts to move by each barcode pair is given by $n_{\text{steps}}=N_{\text{steps}}/B$ or the following:

$$n_{\text{steps}}\propto f^{0.5}N^{1.5}.$$ [3]

The power law [**3**] suggests that the time to copy connectivity does grow with the network size; however, the growth is described by a power law with the relatively small exponent of 1.5. Thus, cloning a network with 10 times more neurons is expected to take about 30 times more time.

In our study, we derived several results on theoretical plausibility of copying the structure of biological neuronal networks. We were motivated by the conventional assumption that neuronal network connectivity carries an imprint of long-term memory and, as such, is an essential substrate of network function (23). Copying (cloning) connectivity might facilitate the transfer of these imprints from one biological network to another. To model network formation, we used a formalism based on the cost function, which is found useful in explaining formation of networks during neural development (8, 16–18). Since our focus was on theoretical plausibility, we did not explore the biological mechanism that could implement the cost function used in this study. Such biological mechanism has to be fairly intricate, as, to form OBOC solutions, barcodes have to agglomerate into the same cell based on their identity defined by their sequence. Precedents to OBOC solution can be found in systems implementing gene expression, for example. Thus, in the olfactory system, each olfactory sensory neuron chooses to express a single olfactory gene out of thousands of possibilities (24). It seems, therefore, that implementations of OBOC solution might involve a system based on gene expression. Further studies are needed to explore these potential mechanisms both experimentally and theoretically.

Our approach can be extended in several directions. For example, in our model, connections were binary and each binary synapse was encoded by a single pair of barcodes. Multiple levels of synaptic strengths can be introduced into our approach by using more than one barcode pair per synaptic connection. A connection encoded by several barcode pairs might be stronger due to the formation of multiple disconnected synaptic active zones between a pair of cells or an increased synaptic area within a single active zone. Although our model cannot distinguish between these two mechanisms, both of them lead to an overall increase in the synaptic conductance between two cells, and, consequently, their effects are similar. In the model explored here, the total number of neurons matched the number of barcode types. This assumption was made to simplify our analysis. It is almost certain to fail in realistic cases. Finally, it is tempting to expand our approach by including multiple sets of barcodes that implement a hierarchy of connection rules. One set of barcodes pairs might encode connectivity between brain regions, while different barcode sets may enforce the rules on mesoscopic and microscopic scales. Including multiple levels of connectivity rules could accelerate wiring large-scale networks. These extensions of our approach could be further investigated.

## Methods

We generated random directed target networks of various topologies, sizes N, and connectivity densities f, as described below. We used 10 different network sizes from N=10 to 10,000 cells, spaced exponentially. In the case of Erdős–Rényi (19) networks, for each network size, we used 10 different connectivity densities from f=0.05 to f=0.80, spaced exponentially, 15 samples each. In the case of scale-free (Barabási–Albert) (20) networks, connectivity densities from $f=10^{-3}$ to f=0.5 were defined by the network structure. These networks were defined by the set of barcode pairs. In the group of simulations with fixed synapses, we introduced these barcode pairs into the fully connected tabula rasa network. We then relocated the barcode pairs using the MMC process, which stochastically minimizes the cost function [**1**]. At the end of simulation, we removed synapses carrying no barcode pairs. In the group of simulations with dynamic synapses, synapses were created and eliminated at the same time as the barcodes were moved between them. In this case, tabula rasa network was random and sparse, with the sparseness parameter $f_{\text{TR}}=f+0.3\cdot \left(1-f\right)$. Here, f is barcode connectivity sparseness, as before. This network had random Erdős–Rényi structure. Initially, we populated tabula rasa network with barcode pairs randomly. Synaptic network had therefore an excess of $0.3\cdot \left(1-f\right)N^{2}$ synapses over what is needed to form an OBOC state. These empty (barcodeless) synapses can therefore be used as relocation targets for the barcode pairs. Empty synapses were pruned and reassigned randomly every $N_{a}$ attempts to move a barcode. $N_{a}$ was adjusted in such a way that all empty synapses are reassigned on average 100 times during a simulation. This parameter was intended to approximate synaptic turnover during neural development (8, 9). A simulation was terminated when OBOC solution was achieved. All of our simulations converged to the OBOC solution within the number of steps equal to 100 times the average. At the end of the simulation, empty synapses $\left[0.3\cdot \left(1-f\right)N^{2}\right]$ are eliminated, similarly to the case of fixed synapses.

For both the cases of fixed and dynamic synapses, barcodes were relocated between synapses via jumps, swaps and flips as described, according to MMC statistical rules. The probabilities of attempting these three operations were 1−f, f−1/N, and 1/N, respectively. During each of the operations, barcodes were inserted in a random orientation. Multiple barcodes were allowed to reside in a single synapse at a time. Our theory (Theorem 2) indicates that jumps alone are sufficient for recreation of the target connectivity; however, we included two other types of the movements for the sake of generality. We assigned the probabilities of attempting a jump, a swap, or a flip so that it can be viewed as swap between two random synapses, with at least one of them occupied by a barcode pair. Thus, the probability of attempting a jump is 1−f, that is, the probability of another synapse to be empty. The probability of a flip is 1/N, that is, equal to the likelihood of selecting the same synapse twice. Because swaps and flips are very computationally inefficient, for the simulations of networks with $N=10^{4}$, we did not attempt any swaps or flips. We observed similar rates of convergence, in this case suggesting that swaps are not that important for convergence, as hinted by Theorem 2.

We used γ=2 and ε=10 in our simulations. To minimize the cost function [**1**], we used MMC procedure (8, 16–18). The value of temperature was chosen to be sufficiently low $\left(T=10^{-4}\right)$ to yield convergence of the algorithm to the correct solution. Each simulation only terminated when an OBOC solution was achieved. Due to *Theorem 2*, connectivity copying can be accomplished via a greedy algorithm. We used MMC procedure because it is quantitatively close to the processes guiding the formation of synaptic connectivity (8, 16–18). To compare connectivity on each step to the target connectivity, we used a greedy procedure that finds dominant barcodes for each cell. To quantify the speed of the convergence, for each MMC simulation, we computed the number of attempts to move the barcodes before a perfect OBOC solution was achieved (Fig. 4). We used linear regression in log–log space to approximate the number of steps to convergence.

## Appendix

### Theorem 1.

*Let* ${\hat{W}}_{B}$ *be the target connectivity defined by the barcode pairs*. *Let* ${\hat{W}}_{N}$ *be a cell connectivity corresponding to an OBOC solution for the same set of the barcode pairs arising after barcode-free synapses are eliminated*. *Then*, *a one-to-one mapping* $\hat{M}$ *exists between the set of barcodes and the neurons*, *which makes* ${\hat{W}}_{B}=\hat{M}{\hat{W}}_{N}{\hat{M}}^{T}$.

#### 

##### Proof:

Although this theorem is somewhat trivial, we prove it here for completeness. Because, in our approach, the number of barcodes is equal to the number of cells, in OBOC solution, every cell has a unique barcode. One can thus use barcodes to identify cells. We then define a permutation matrix $\hat{P}$ that determines the assignment of barcodes into cells in the OBOC solution. An entry $P_{\alpha n}$ in this matrix is equal to 1 if barcode α is present in cell n and zero otherwise. By the property of permutation matrix $\hat{P}{\hat{P}}^{T}=\hat{P}{\hat{P}}^{-1}=\hat{I}$. Clearly, ${\hat{W}}_{B}=\hat{P}{\hat{W}}_{N}{\hat{P}}^{T}$, and ${\hat{W}}_{N}={\hat{P}}^{T}{\hat{W}}_{B}\hat{P}$, which proves the theorem, if $\hat{M}\equiv \hat{P}$.

### Theorem 2.

*For* γ=2 *and* ε≥1, *in a non-OBOC state*, *there is always a barcode jump decreasing the cost function*.

#### 

##### Proof:

According to Eq. **1** of the main text, if barcode of the type β is relocated from the cell number m to the cell number n, the change in the cost function ΔH is given by the following:

$$\Delta H_{m\to n}=c_{m\beta }-c_{n\beta }-1+\varepsilon \sum_{\alpha \neq \beta }\left(c_{n\alpha }-c_{m\alpha }\right).$$ [4]

##### Case 1 (each barcode is contained in one cell only):

Assume that the barcodes are in a non-OBOC state. The simplest case is when each barcode type is located in a single neuron. In this case, several barcode types can share the same neuron, leading to the non-OBOC state. Since the number of barcodes is equal to the number of cells, this implies that the network contains a cell with no barcodes. We denote a cell hosting multiple barcode types by index m and an empty one by index n. The barcode type with the minimal abundance in m is called β. Because $c_{n\alpha }=0$ for any α, the change in cost function for relocating the barcode β to the empty cell n is as follows:

$$\Delta H_{m\to n}=c_{m\beta }-1-\varepsilon \sum_{\alpha \neq \beta }c_{m\alpha }.$$ [5]

Because ε≥1, we have $\Delta H_{m\to n}\leq -1<0$.

##### Case 2 (at least one barcode is shared between two cells):

In this case, we can pick a pair of cells m and n, both hosting at least one copy of the barcode of the same type, henceforth referred to as β. Using Eq. **4**, we can compute the sum of changes in the cost function for opposite movements of the barcode, that is, from m to n and from n to m:

$$\Delta H_{m\to n}+\Delta H_{n\to m}=-2<0.$$ [6]

It means that at least one of those two cost-function changes is negative. Thus, we have shown, that in all possible non-OBOC states, given γ=2 and ε≥1, there is at least one possible movement that decreases the cost function.

### Corollary.

*For* γ=2 *and* ε≥1, *all of the minima of the cost function* [**1**] *correspond to OBOC solutions*.

#### 

##### Proof:

Assume we are in a cost-function minimum, which is non-OBOC. According to *Theorem 2*, there is a barcode pair movement, decreasing the cost function. Therefore, it is not a minimum.

### Theorem 3.

*For* γ=2 *and* ε≥1, *convergence to a minimum of the cost function* [**1**] *takes a number of steps limited from above by a number polynomial in the number of network nodes (cells)*.

#### 

##### Proof:

The cost-function spectrum is discrete and limited. For example, if ε is a natural number, then the cost function is an integer number. The lower boundary of the cost-function spectrum corresponds to an OBOC solution (*Corollary*), and equals to $-f^{2}N^{3}$. As we start with a random distribution of the barcodes, the higher boundary approximately equals to $\varepsilon f^{2}N^{3}$. Therefore, the number of successful steps to the convergence cannot exceed $\left(1+\varepsilon \right)f^{2}N^{3}$. In every non-OBOC state, there is at least one in $fN^{4}$ barcode jumps, decreasing the cost function (*Theorem 2*). Thus, the overall number of the steps to convergence does not exceed $\left(1+\varepsilon \right)f^{3}N^{7}$, which is polynomial.
